# LEAP2 Impairs the Capability of the Growth Hormone Secretagogue Receptor to Regulate the Dopamine 2 Receptor Signaling

**DOI:** 10.3389/fphar.2021.712437

**Published:** 2021-08-10

**Authors:** Emilio R. Mustafá, Santiago Cordisco González, Marjorie Damian, Sonia Cantel, Severine Denoyelle, Renaud Wagner, Helgi B. Schiöth, Jean-Alain Fehrentz, Jean-Louis Banères, Mario Perelló, Jesica Raingo

**Affiliations:** ^1^ Laboratory of Electrophysiology of the Multidisciplinary Institute of Cell Biology [IMBICE, Argentine Research Council (CONICET) and Scientific Research Commission, Province of Buenos Aires (CIC-PBA), National University of La Plata (UNLP)], La Plata, Argentina; ^2^ Institut des Biomolécules Max Mousseron (IBMM), Université Montpellier, CNRS, Montpellier, France; ^3^ Plateforme IMPReSs, CNRS UMR7242, Biotechnologie et Signalisation Cellulaire, École Supérieure de Biotechnologie de Strasbourg, Strasbourg, France; ^4^ Department of Neuroscience, Uppsala University, Uppsala, Sweden; ^5^ Institute for Translational Medicine and Biothechnology, I. M. Sechenov First Moscow State Medical University, Moscow, Russia; ^6^ Laboratory of Neurophysiology of the Multidisciplinary Institute of Cell Biology [IMBICE, Argentine Research Council (CONICET) and Scientific Research Commission, Province of Buenos Aires (CIC-PBA), National University of La Plata (UNLP)], La Plata, Argentina

**Keywords:** GPCR, heterodimerization, Cav2.2, ghrelin receptor, dopamine receptor, constitutive activity

## Abstract

The growth hormone secretagogue receptor (GHSR) signals in response to ghrelin, but also acts via ligand-independent mechanisms that include either constitutive activation or interaction with other G protein-coupled receptors, such as the dopamine 2 receptor (D2R). A key target of GHSR in neurons is voltage-gated calcium channels type 2.2 (Ca_V_2.2). Recently, the liver-expressed antimicrobial peptide 2 (LEAP2) was recognized as a novel GHSR ligand, but the mechanism of action of LEAP2 on GHSR is not well understood. Here, we investigated the role of LEAP2 on the canonical and non-canonical modes of action of GHSR on Ca_V_2.2 function. Using a heterologous expression system and patch-clamp recordings, we found that LEAP2 impairs the reduction of Ca_V_2.2 currents induced by ghrelin-evoked and constitutive GHSR activities, acting as a GHSR antagonist and inverse agonist, respectively. We also found that LEAP2 prevents GHSR from modulating the effects of D2R signaling on Ca_V_2.2 currents, and that the GHSR-binding N-terminal region LEAP2 underlies these effects. Using purified labeled receptors assembled into lipid nanodiscs and Forster Resonance Energy Transfer (FRET) assessments, we found that the N-terminal region of LEAP2 stabilizes an inactive conformation of GHSR that is dissociated from Gq protein and, consequently, reverses the effect of GHSR on D2R-dependent Gi activation. Thus, our results provide critical molecular insights into the mechanism mediating LEAP2 modulation of GHSR.

## Introduction

The growth hormone secretagogue receptor (GHSR) is a G protein-coupled receptor (GPCR) highly expressed in the brain (Muller et al., 2015; Cornejo et al., 2020). GHSR regulates key physiological functions including appetite, neuroendocrine axis, autonomic nervous system activity and complex cognitive functions, such as reward-related behaviors (Muller et al., 2015; Cornejo et al., 2020). The effects of GHSR are mainly attributed to its regulation of neuronal activity (Shi et al., 2013; Ribeiro et al., 2014; Ghersi et al., 2015; Lee et al., 2016). The first described endogenous ligand for GHSR is ghrelin, a peptide hormone mainly produced in the stomach (Kojima et al., 1999). Ghrelin is a GHSR agonist that triggers signaling through different pathways involving Gq, Gi/o, G12/13 and arrestins (M'Kadmi et al., 2015; Mende et al., 2018). Further studies showed that GHSR also acts via several ghrelin-independent mechanisms.

**GRAPHICAL ABSTRACT:**
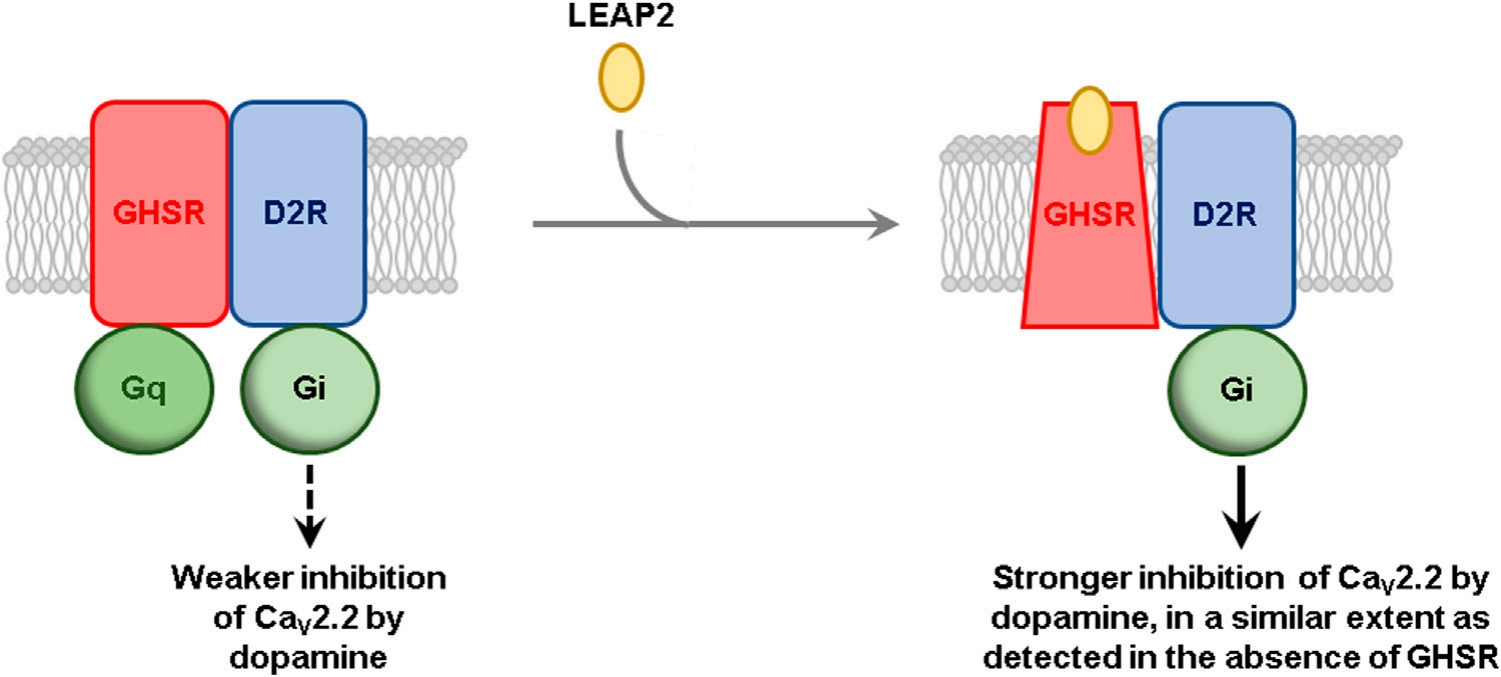
**LEAP2 Proposed model for the effect of LEAP2 on GHSR-D2R-mediated inhibition of CaV2.2**. In the absence of LEAP2, GHSR is preassembled to Gq protein and in an active-like conformation due to its high constitutive activity, which impairs D2R-mediated inhibition of CaV2.2. LEAP2 stabilizes an inactive conformation of GHSR that rearranges the geometry of the GHSR-D2 heteromer and dissociates pre-assembled Gq protein, leading to a stronger inhibition of CaV2.2, in a similar extent as detected in the absence of GHSR. For the sake of simplicity, heterotrimeric G proteins are shown as a single shape.

In the absence of ghrelin, GHSR can adopt an inactive G protein-pre-assembled conformation or an active conformation (Damian et al., 2015). This ligand-independent active state of the receptor induces constitutive GHSR activity, which activates Gq protein to ∼50% of its maximal capacity *in vitro* (Holst et al., 2003). Constitutive GHSR activity has been suggested to have physiological consequences in rodent models and in humans (Pantel et al., 2006; Fernandez et al., 2018; Torz et al., 2020). Furthermore, GHSR can form heteromers with other receptors enabling mutual allosteric regulations that affect each signaling cascade and also allows cross-talk between the signaling pathways of each receptor (Hedegaard and Holst, 2020). The interaction of GHSR with the dopamine type 2 receptor (D2R) has dramatic physiological implications: GHSR knockout mice fail to decrease food intake in response to cabergoline, a potent D2R agonist (Kern et al., 2012). GHSR-D2R interaction in lumbosacral autonomic neurons also appear to regulate dopamine effects on the defecation pathways (Furness et al., 2020). The GHSR-D2R interaction shifts the dopamine-evoked signaling of D2R from a canonical (Beaulieu and Gainetdinov, 2011) to a non canonical Gi/o protein signaling, in a ghrelin-independent manner that involves Gβγ subunits (Kern et al., 2012). Accordingly, GHSR affects the kinetics of D2R-mediated Gi activation via Gαi conformational dynamics in an *in vitro* isolated system (Damian et al., 2018). Thus, GHSR acts via multiple mechanisms with distinct functional roles.

The liver-expressed antimicrobial peptide 2 (LEAP2) was identified as a new endogenous ligand for GHSR (Ge et al., 2018). LEAP2 is a peptide synthesized by endocrine cells of the liver and the intestinal tract (Krause et al., 2003). In rodents, LEAP2 impairs the hyperglycemic and orexigenic effects of ghrelin (Ge et al., 2018). In hypothalamic neurons, LEAP2 impairs the depolarizing actions of ghrelin (Mani et al., 2019). LEAP2 acts as an antagonist of GHSR that blocks ghrelin-evoked Gq protein signaling (Ge et al., 2018; M'Kadmi et al., 2019; Wang et al., 2019). In GHSR-transfected cells, LEAP2 suppresses the constitutive activation of Gq and G13 proteins, acting as a GHSR inverse agonist (Barrile et al., 2019; M'Kadmi et al., 2019). The bioactive portion of LEAP2 resides at the N-terminal region of the peptide, which binds to GHSR and impairs both ghrelin-evoked and constitutive signaling pathways (M'Kadmi et al., 2019). Thus, GHSR activity is regulated by at least two endogenous ligands, ghrelin and LEAP2, that display opposite actions (Cornejo et al., 2021).

GHSR regulates voltage-gated calcium channels (Ca_V_), which has diverse impacts on neuronal activity. Ghrelin-evoked GHSR activity inhibits presynaptic Ca_V_2 currents in neurons, favoring a reduction of GABA release and a subsequent activation of postsynaptic neurons (Lopez Soto et al., 2015; Cabral et al., 2016; Torz et al., 2020). Ghrelin-evoked GHSR activation also inhibits somato-dendritic Ca_V_3.3 (Mustafa et al., 2020). Notably, constitutive GHSR activity inhibits the forward traffic of Ca_V_2 to the plasma membrane in GABA neurons and reduces basal calcium-dependent inhibitory neurotransmission (Lopez Soto et al., 2015; Mustafa et al., 2017; Martinez Damonte et al., 2018; Torz et al., 2020). Interestingly, GHSR-D2R heteromer shows increased basal inhibition of Ca_V_2.2 currents compared to GHSR alone, as well as decreased dopamine-induced inhibition of Ca_V_2.2 compared to D2R alone (Cordisco Gonzalez et al., 2020). Thus, LEAP2 could potencially affect GHSR regulation of Ca_V_ via a variety of mechanisms.

Here, we confirmed that LEAP2 impairs both ghrelin-dependent and ghrelin-independent GHSR inhibition of Ca_V_2.2. Next, we tested the hypothesis that LEAP2 affects the ability of GHSR to modulate the action of D2R on Ca_V_2.2. We found that this indeed occurs and that N-terminal end of LEAP2 was sufficient to the effect. Moreover, we found that LEAP2 affects GHSR-D2R heteromer conformation and their coupling to G proteins *in vitro*.

## Materials and Methods

### Cell Culture and Transient Transfections

HEK293T cells were grown in Dulbecco’s modified Eagle’s medium (DMEM; Gibco) with 10% fetal bovine serum (Internegocios) and transfected with plasmids containing Ca_V_2.2 (#AF055477), auxiliary subunits Ca_V_β_3_ (#M88751) and Ca_V_α_2_δ_1_ (#AF286488), GFP-containing plasmid (to identify transfected cells) with or without GHSR-containing plasmid (#AY429112) and/or D2R-containing plasmid (MG226860-Origene). Cells were transfected using Lipofectamine 2000 (Invitrogen) and Opti-MEM (Gibco). For patch-clamp, cells were treated with 0.25 mg/ml trypsin (Microvet), rinsed twice and kept at room temperature (RT) in DMEM.

### Drugs

Ghrelin was purchased from Global Peptide (PI-G-03), [DArg1,D-Phe5,D-Trp7,9,Leu11]-substance P analogue (SPA) from Santa Cruz (sc-361166), Dopamine hydrochloride from Sigma-Aldrich (H8502) and LEAP2 from Phoenix Pharmaceutical (T-075-40). JMV2959 was synthesized as described (Moulin et al., 2007). LEAP2 region peptides were synthesized, purified by RP-HPLC and characterized by LC-MS and MALDI-MS/MS (>95% purity) (M'Kadmi et al., 2019).

### Electrophysiology

Whole-cell patch-clamp recordings in voltage-clamp configuration were performed using Axopatch 200 amplifier (Molecular Devices). Data were sampled at 20 kHz and filtered at 10 kHz (−3 dB) using PCLAMP8.2.0.235 software (Molecular Devices). Recording pipettes (2–4 MΩ) were filled with internal solution (in mM): 134 CsCl, 10 EGTA, 1 EDTA, 10 HEPES and 4 MgATP (pH 7.2 with CsOH). Leak current was subtracted online using a P/-4 protocol. External solution was perfused (flow rate ∼1 ml/min) by gravity and contained (in mM): 140 choline chloride, 10 HEPES, 1 MgCl_2_.6H_2_O and 2 CaCl_2_.2H_2_O (pH 7.3–7.4 with CsOH). Recordings were obtained at RT.

### Protein Preparation

GHSR-D2R heteromer in lipid nanodiscs was prepared as described in Damian et al. (2018) with the exception that DIBMA (Anatrace) was used instead of SMA to solubilize the receptor-containing liposomes. G proteins were produced as described in Damian et al. (2012). 5HW was incorporated in Gα_i1_ during bacterial expression using the CY(DE3)pLysS *E. coli* strain (Oliveira-Souza et al., 2017). Labeling of GHSRC304^7.34^ with Lumi-4 Tb on the reactive cysteine C304^7.34^ was done by incubating the purified receptor in A8-35, *i.e.,* before insertion into the liposomes, with the Lumi-4 Tb maleimide dye at 4°C for 16 h (1:5 protein-to-dye molar ratio). For intramolecular FRET measurements, GHSR with a TAG amber codon at the position encoding F71^1.60^ and a single reactive cysteine at position 255^6.27^ was produced and labeled with Click-IT Alexa Fluor 488 DIBO Alkyne (LifeTechnologies) and Alexa Fluor 350 maleimide (ThermoFisher) before insertion into the liposomes, *i.e*., in its A8-35-stabilized state (Damian et al., 2015). Labeling of Gα_q_ and Gα_i1_ on their N-terminus with AF-350 or AF-488 was carried out using the NHS derivative of the fluorophore (ThermoFisher) at neutral pH (Damian et al., 2015).

### Homogenous Time-Resolved Fluorescence Assays

For HTRF-monitored GHSR-D2R dimerization assays, GHSR labeled with Lumi4-Tb on C304^7.34^ was used as the donor and an XL255-labeled anti-Flag M2 antibody (CisBio) bound to the Flag-tag of the D2R as the acceptor (Damian et al., 2018). Fluorescent signals were measured at 620 nm (emission of the Tb donor) and 665 nm (FRET signal) using a Cary Eclipse spectrofluorimeter (Varian).

### FRET Measurements

Fluorescence emission spectra were recorded at 20°C on a Cary Eclipse spectrofluorimeter exciting AF-350 at 347 nm or AF-488 at 500 nm. The receptors (0.5 μM) and the ligands (10 μM) were incubated 30 min at RT before spectroscopic measurements. Buffer contributions were subtracted. The proximity ratio was calculated from the emission spectra as described (Granier et al., 2007).

### Gi Activation Assays

Association of GTPγS to Gi was carried out using the fluorescence properties of 5HW introduced in the Gα_i1_ subunit (Damian et al., 2018). Reaction conditions (in mM) were: 0.0001 GDP-bound Gα_q_β_1_γ_2_ and Gα_i1_β_1_γ_2_, and 0.00002 receptor in lipid nanodiscs in a buffer containing 20 HEPES, 130 NaCl, and 5 MgCl_2_ (pH 7.5) The receptors were first incubated with different ligands (10 µM) before the G proteins addition. The rate of GTPγS binding to Gα_i1_ was determined by monitoring the relative increase in the intrinsic 5HW fluorescence (λ_exc_: 315 nm; λ_em_: 350 nm) as a function of time (1 data point/10 s for 1,800 s) after the addition of GTPγS using the RX2000 Rapid Kinetics accessory (Applied Photophysics) of the spectrophotometer. The increase in 5HW fluorescence was fitted with a pseudo first-order exponential association model to derive the apparent activation rate constant.

### Statistics

Data were analyzed and visualized using the Prism 6 (GraphPad Software, Inc.). When the sample size allowed it, data normality was tested using D’Agostino and Pearson test. Data with normal distribution were compared with Student’s unpaired t tests or regular one-way ANOVA with Tukey’s post-test, depending on the number of groups. When a normal distribution was not found or could not be tested due to the small sample size, data were compared with Kruskal-Wallis test and Dunn’s post-test. Data are displayed as mean ± se and the tests used for each comparison are indicated in the figure legends.

## Results

**FIGURE 1:**
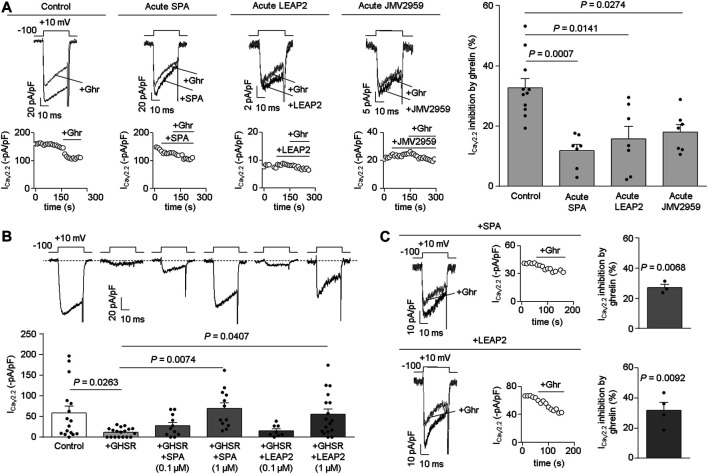
Acute LEAP2 reduces ghrelin-dependent and -independent effects of GHSR on Ca_V_2.2 currents. **(A)** Representative traces and time courses **(left)** of Ca_V_2.2 current (I_CaV2.2_) from HEK293T cells cotransfected with Ca_V_2.2, Ca_V_β_3_, Ca_V_α_2_δ_1_ and GHSR in 0.1 GHSR:Ca_V_2.2 molar ratio in control condition and ghrelin (+Ghr) application (Control, *n* = 11); or consecutive SPA and ghrelin application (Acute SPA, *n* = 7); or consecutive LEAP2 and ghrelin application (Acute LEAP2, *n* = 7); or consecutive JMV2959 and ghrelin application (Acute JMV2959, *n* = 7). Bars **(right)** represent averaged I_CaV2.2_ inhibition by 0.5 µM ghrelin application for each condition. Statistical significance was evaluated by Kruskal-Wallis and Dunn’s post-test (vs. Control). **(B)** Representative traces **(top left)** of Ca_V_2.2 current (I_CaV2.2_) from HEK293T cells cotransfected with Ca_V_2.2, Ca_V_β_3_, Ca_V_α_2_δ_1_ and, empty pcDNA3.1 (Control, *n* = 16) or GHSR in 0.6 GHSR:Ca_V_2.2 molar ratio (+GHSR, *n* = 18), pre-incubated or not with 0.1 µM or 1 µM of SPA [+GHSR +SPA (0.1 µM), *n* = 11; +GHSR +SPA (1 µM), *n* = 12], or 0.1 µM or 1 µM of LEAP2 [+GHSR +LEAP2 (0.1 µM), *n* = 8; +GHSR+ LEAP2 (1 µM), *n* = 17] during 20 h. Bars **(bottom left)** represent averaged I_CaV2.2_ levels for each condition. Statistical significance was evaluated by One Way ANOVA and Tukey’s post-test. **(C)** Representative traces and time courses of I_CaV2.2_ from HEK293T cells cotransfected with Ca_V_2.2, Ca_V_β_3_, Ca_V_α_2_δ_1_ and GHSR in 0.6 GHSR:Ca_V_2.2 molar ratio pre-incubated with 1 µM of SPA **(top right)** or with 1 µM of LEAP2 **(bottom right)** during 20 h. Ghrelin was applied after washing SPA (SPA, *n* = 3) or LEAP2 (LEAP2, *n* = 4). Bars represent averaged I_CaV2.2_ inhibition by 0.5 µM ghrelin application for each condition. Statistical significance was evaluated by One-Sample Student’s *t* test, test value = 0. The test-pulse protocol consisted in square pulses applied from −100 to +10 mV for 30 ms every 10 s.

**LEAP2 impairs ghrelin-evoked GHSR inhibition of Ca_V_2.2 currents.** In order to test if LEAP2 blocks ghrelin-evoked GHSR inhibition of Ca_V_2.2 currents, we performed patch-clamp recordings in HEK293T cells co-expressing Ca_V_2.2 (plus its auxiliary subunits) and GHSR. We found that pre-treatment with LEAP2 (∼1 min, 0.5 µM) reduced the inhibition of Ca_V_2.2 currents induced by equimolar concentration of ghrelin (0.5 µM) (Pantel et al., 2006; Mustafa et al., 2020), similar to [DArg1,D-Phe5,D-Trp7,9,Leu11]-substance P analogue (SPA) pre-treatment (∼1 min, 0.5 µM) (Figure 1A). Additionally, we confirmed that JMV2959 (∼1 min, 50 µM), a well-described GHSR antagonist (M'Kadmi et al., 2015), reduced the ghrelin-induced Ca_V_2.2 inhibition. Notably, the acute application of LEAP2, SPA or JMV2959 failed to affect Ca_V_2.2 currents in the absence of ghrelin [I_CaV2.2_ inhibition (%) by: LEAP2 = 17.93 ± 6.50, *n* = 4, *p* = 0.0704; SPA = 8.67 ± 3.69, *n* = 4, *p* = 0.1007; JMV2959 = 12.19 ± 6.67, *n* = 4, *p* = 0.1651; One-Sample Student’s *t* tests versus zero]. Thus, LEAP2, JMV2959 and SPA impair ghrelin-evoked GHSR inhibition of Ca_V_2.2.

**LEAP2 impairs the effect of constitutive GHSR activity on Ca_V_2.2 current.** Next, we tested the effect of LEAP2 on basal Ca_V_2.2 currents in HEK293T cells transfected with GHSR and Ca_V_2.2. We used a GHSR/Ca_V_2.2 molar ratio (0.6) sufficient to reduce basal Ca_V_2.2 currents (Lopez Soto et al., 2015). Cells were cultured in medium alone or containing LEAP2 or SPA (0.1 and 1 µM, respectively) for 20 h, after which calcium currents were recorded. Overnight treatment with 1 µM LEAP2 significantly impaired the basal reduction of Ca_V_2.2 current induced by GHSR co-expression whereas 0.1 µM LEAP2 was insufficient to occlude the basal GHSR effect (Figure 1B). Similarly, 1 µM SPA impaired the basal reduction of Ca_V_2.2 current induced by GHSR co-expression, as previously shown by our group (Lopez Soto et al., 2015; Mustafa et al., 2017; Cordisco Gonzalez et al., 2020). Basal Ca_V_2.2 currents were unaffected by overnight incubation with JMV2959, which lacks GHSR inverse agonist activity (M'Kadmi et al., 2015) (−5.27 ± 2.60 pA/pF, *n* = 8, *p* = 0.0822, One-Sample Student’s *t* test versus zero). Additionally, we tested whether acute application of ghrelin (0.5 µM) modulates Ca_V_2.2 in GHSR-expressing cells that were incubated overnight with LEAP2 or SPA (1 µM) and washed. Ghrelin inhibited Ca_V_2.2 currents, regardless of overnight incubation with LEAP2 or SPA (Figure 1C), as previously shown for SPA (Lopez Soto et al., 2015). Thus, pre-treatment with LEAP2 impairs the effect of GHSR on basal Ca_V_2.2 currents, suggesting that it is a GHSR inverse agonist.

**FIGURE 2:**
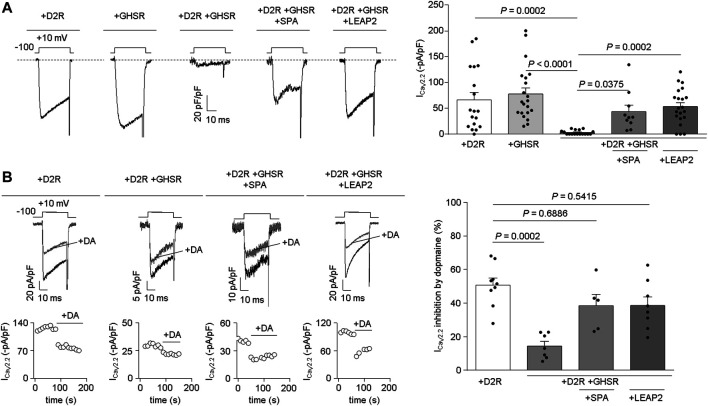
LEAP2 impairs the basal reduction of Ca_V_2.2 currents by GHSR and D2R coexpression and LEAP2 ameliorates the ability of GHSR to impair dopamine-induced inhibition of Ca_V_2.2 currents. **(A)** Representative traces **(left)** of Ca_V_2.2 current (I_CaV2.2_) from HEK293T cells cotransfected with Ca_V_2.2, Ca_V_β_3_, Ca_V_α_2_δ_1_ and either D2R (+D2R, *n* = 18), GHSR (+GHSR, *n* = 21) or GHSR and D2R (+D2R +GHSR, *n* = 17) pre-incubated or not with 1 µM SPA (+D2R +GHSR +SPA, *n* = 10) or 0.1 µM LEAP2 (+D2R +GHSR +LEAP2, *n* = 21) in a 0.1 GPCR:Ca_V_2.2 molar ratio. Bars **(right)** represent averaged I_CaV2.2_ levels for each condition. Statistical significance was evaluated by Kruskal-Wallis and Dunn’s post-test. **(B)** Representative traces and time courses **(left)** of Ca_V_2.2 current (I_CaV2.2_) from HEK293T cells cotransfected with Ca_V_2.2, Ca_V_β_3_, Ca_V_α_2_δ_1_ and D2R (+D2R, *n* = 9) or GHSR and D2R pre-incubated or not (+D2R +GHSR, *n* = 7) with 1 µM SPA (+D2R +GHSR +SPA, *n* = 5) or 0.1 µM LEAP2 (+D2R +GHSR +LEAP2, *n* = 8) in control condition and after dopamine application (10 µM, +DA); 0.1 GPCR:Ca_V_2.2 molar ratio. Bars **(right)** represent averaged I_CaV2.2_ inhibition by 10 µM dopamine application for each condition. Statistical significance was evaluated by Kruskal-Wallis and Dunn’s post-test (versus +D2R). The test-pulse protocol consisted in square pulses applied from −100 to +10 mV for 30 ms every 10 s.

**LEAP2 prevents GHSR from modulating the effects of D2R on Ca_V_2.2 currents.** We tested if LEAP2 affects GHSR-D2R heteromer reduction of basal Ca_V_2.2 currents. We recorded basal Ca_V_2.2 currents in HEK293T cells transfected with D2R, GHSR or GHSR-D2R (GPCR:Ca_V_2.2 molar ratio: 0.1) and confirmed that co-expression of GHSR and D2R reduces basal Ca_V_2.2 currents (Figure 2A). Overnight treatment with 1 µM SPA of cells co-expressing GHSR and D2R restored Ca_V_2.2 currents to control levels (D2R- or GHSR-expressing cells) as previously reported (Cordisco Gonzalez et al., 2020). Interetingly, 0.1 µM LEAP2 was sufficient to have an effect comparable to 1 µM SPA (Figure 2A). We discarded an effect of LEAP2 on Ca_V_2.2 currents in D2R-expressing cells alone (−41.42 ± 13.40 pA/pF, *p* > 0.9999, Kruskal-Wallis and Dunn’s post-test versus +D2R). Thus, LEAP2 blocks the GHSR-D2R co-expression effects on Ca_V_2.2 currents.

Next, we explored whether LEAP2 affects the GHSR-mediated impairment of dopamine-evoked D2R inhibition of Ca_V_2.2 currents. Dopamine (10 μM) induced a ∼56% inhibition of Ca_V_2.2 currents in D2R-expressing cells, and this effect was significantly reduced in D2R-GHSR expressing cells as expected (Cordisco Gonzalez et al., 2020). Overnight treatment with LEAP2 restored the dopamine-evoked inhibition of Ca_V_2.2 currents under these conditions, similar to treatment with SPA (Figure 2B). LEAP2 pretreatment thus impairs the effect of GHSR co-expression on dopamine-evoked inhibition of Ca_V_2.2 by D2R.

**FIGURE 3:**
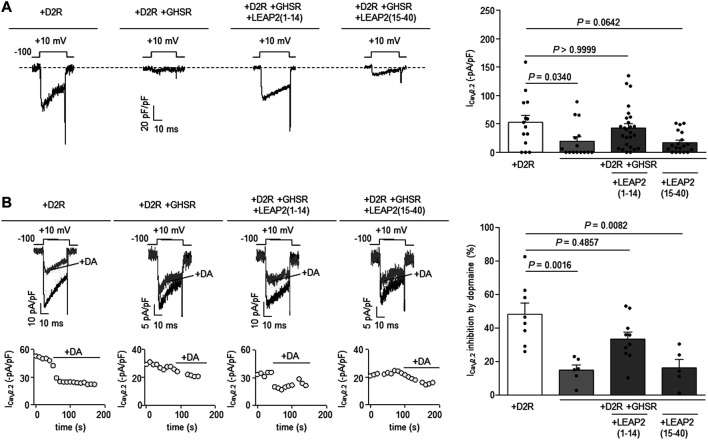
LEAP2 (1-14) impairs GHSR modulation of D2R signaling. **(A)** Representative traces **(left)** of Ca_V_2.2 current (I_CaV2.2_) from HEK293T cells cotransfected with Ca_V_2.2, Ca_V_β_3_, Ca_V_α_2_δ_1_ and either D2R (+D2R, *n* = 14) or GHSR and D2R (+D2R +GHSR, *n* = 16) pre-incubated or not with 0.1 µM LEAP2 (1–14) [+D2R +GHSR +LEAP2 (1–14), *n* = 26] or 0.1 µM LEAP2 (15–40) [+D2R +GHSR +LEAP2 (15–40), *n* = 19] in a 0.1 GPCR:Ca_V_2.2 molar ratio. Bars **(rigth)** represent averaged I_CaV2.2_ levels for each condition. Statistical significance was evaluated by Kruskal-Wallis and Dunn’s post-test (versus +D2R condition). **(B)** Representative traces and time courses **(left)** of Ca_V_2.2 current (I_CaV2.2_) from HEK293T cells cotransfected with Ca_V_2.2, Ca_V_β_3_, Ca_V_α_2_δ_1_ and D2R (+D2R, *n* = 8) or GHSR and D2R pre-incubated or not (+D2R+GHSR, *n* = 6) with 0.1 µM LEAP2 (1–14) [+D2R +GHSR +LEAP2 (1–14), *n* = 10] or 0.1 µM LEAP2 (15–40) [+D2R +GHSR +LEAP2 (15–40), *n* = 5] in control condition and after dopamine application (10 µM, +DA); 0.1 GPCR:Ca_V_2.2 molar ratio. Bars **(right)** represent averaged I_CaV2.2_ inhibition by 10 µM dopamine application for each condition. Statistical significance was evaluated by Kruskal-Wallis and Dunn’s post-test (versus +D2R). The test-pulse protocol consisted in square pulses applied from −100 to +10 mV for 30 ms every 10 s.

**The N-terminal region of LEAP2 is sufficient to impair GHSR modulation of D2R signaling.** We have shown that the LEAP2 N-terminal region binds to GHSR with similar affinity than intact LEAP2 and displays full antagonistic and inverse agonist activities (Cornejo et al., 2019; M'Kadmi et al., 2019). To test if LEAP2 N-terminal region also impairs GHSR modulation of Ca_V_2.2 current inhibition by D2R, we tested a peptide containing the first 14 residues of LEAP2, LEAP2 (1–14). We found that overnight LEAP2 (1–14) treatment of cells co-expressing GHSR-D2R restored Ca_V_2.2 currents to the basal levels found in D2R-expressing cells (Figure 3A). In contrast, overnight treatment with a peptide containing the 25 residues of C-terminal portion of LEAP2, LEAP2 (15–40), did not affect Ca_V_2.2 currents in GHSR-D2R expressing cells (Figure 3A). We also found that pretreatment with LEAP2 (1–14) restored the dopamine-induced inhibition of Ca_V_2.2 currents in cells co-expressing GHSR-D2R, whereas pretreatment with LEAP2 (15–40) have no effect (Figure 3B). Thus, the N-terminal region of LEAP2 is sufficient to impair GHSR modulation of D2R signaling.

**FIGURE 4:**
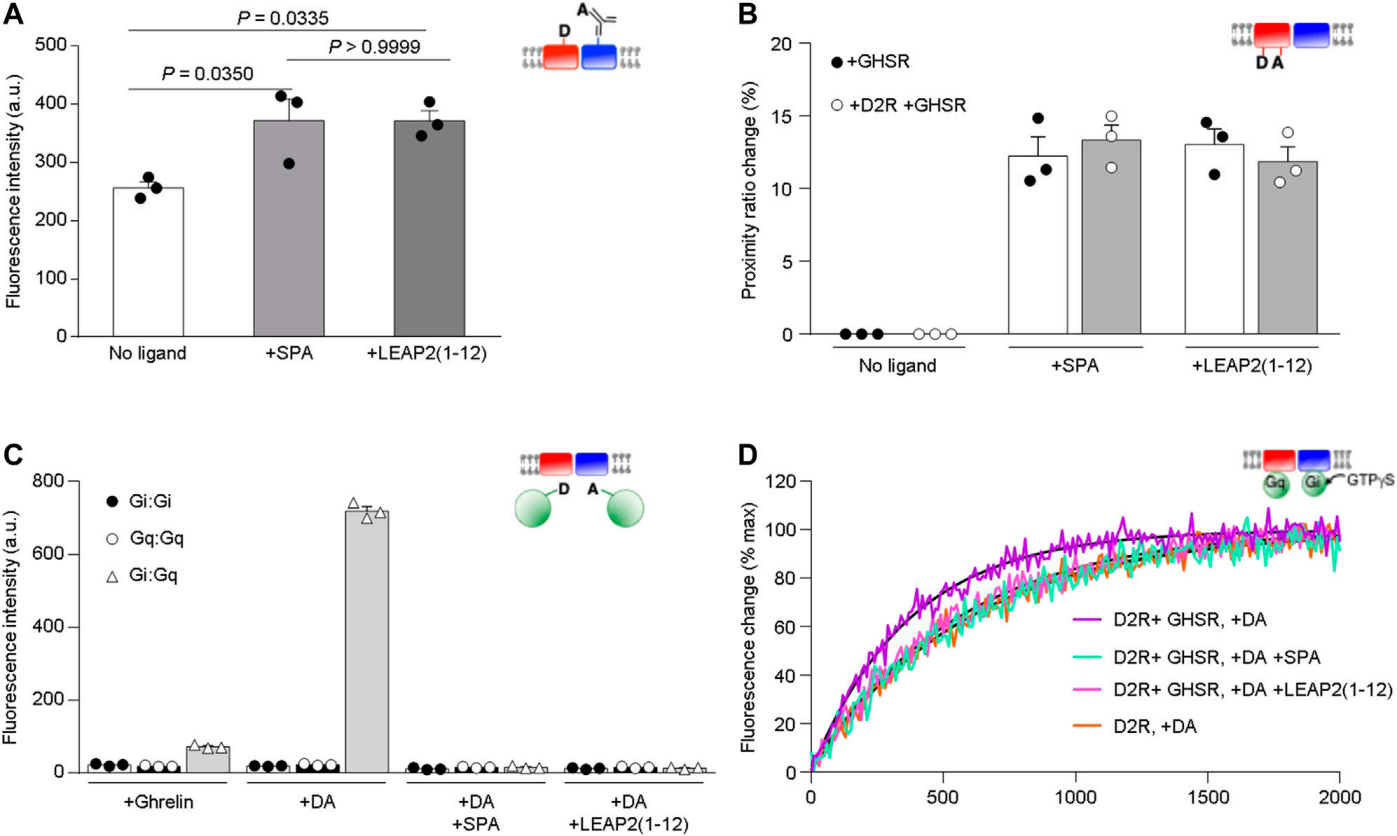
Impact of LEAP2 on GHSR structure and dopamine-mediated Gi activation. **(A)** XL255 emission intensity after Tb-cryptate excitation of proteoliposomes containing Tb-cryptate labeled GHSR and XL255-labeled D2R in absence of ligand (No ligand) or in presence of 10 µM SPA (+SPA) or LEAP2 (1–12) [+LEAP2 (1–12)]. Statistical significance was evaluated by One Way ANOVA and Tukey’s post-test. **(B)** Proximity ratio changes induced by 10 µM of SPA (+SPA) or LEAP2 (1–12) [+LEAP2 (1–12)] calculated from the FRET signal between the fluorophores in TM1 and TM6 of GHSR assembled into lipid nanodiscs either as a homomer (+GHSR) or a heteromer (+GHSR +D2R). **(C)** AF-488 emission intensity after AF-350 excitation. Gαi and Gαq were labeled at their N terminus with AF-350 and AF-488, respectively, and fluorescence was measured in the presence of the labeled G proteins, the GHSR-D2R heteromer in lipid nanodiscs and 10 µM ghrelin (+Ghrelin), 10 µM dopamine (+DA), or 10 µM dopamine in the absence or in the presence of either 10 µM SPA (+DA +SPA) or LEAP2 (1–12) [+DA +LEAP2 (1–12)]. **(D)** GTPγS binding to Gα_i1_ in Gα_i1_β_1_γ_2_ catalyzed by the GHSR-D2R heteromer in the presence of 10 µM dopamine (DA) and in absence or in the presence of either 10 µM SPA or LEAP2 (1–12). GTPγS binding to Gα_i1_ catalyzed under the same conditions by the D2R homomer in the presence of 10 µM dopamine (DA) is given for comparison. The species considered are schematically depicted in all cases (red: GHSR, blue:D2R, green: G protein). Data in (**A**–**C**) is mean ± SD of three experiments.

**The N-terminal region of LEAP2 stabilizes an inactive conformation of GHSR in the D2R-GHSR heteromer and alters dopamine-mediated Gi activation.** We analyzed the effect of the N-terminal region of LEAP2 on the conformational features and functional properties of isolated GHSR-D2R heteromers. First, we monitored the FRET signal between GHSR labeled with a fluorescence donor and D2R labeled with fluorescence acceptor, as this signal reports on the proximity of the two receptors (Damian et al., 2018). The significant FRET signal recorded in the presence of either LEAP2 (1–12) or SPA (Figure 4A) suggests that the binding of these ligands does not trigger major dissociation of the GHSR-D2R heteromer. However, the FRET signal in the presence of LEAP2 (1–12) or SPA was higher than that recorded in the absence of ligands, suggesting that these compounds modify the arrangement of the D2R-GHSR heteromers. Alternatively, such difference in the FRET signal could indicate that the binding of LEAP2 (1–12) affects the dynamics of protomer exchange within the heteromer, as this interaction is a dynamic process (Damian et al., 2018).

Next, we analyzed whether LEAP2 affects the conformational features of GHSR in the heteromer using the intramolecular FRET signal between a fluorescence donor and an acceptor at the cytoplasmic ends of the TM1 and TM6 domains of GHSR, respectively. Labeled GHSR was assembled into lipid nanodiscs with or without unlabeled D2R. We found that LEAP2 (1–12) and SPA significantly changed the intramolecular FRET signal (Figure 4B). Moreover, the proximity ratio change amplitude was unchanged by D2R presence in the lipid nanodisc (Figure 4B) suggesting that LEAP2 (1–12) stabilizes a similar inactive GHSR conformation independently of its interaction with D2R. Under this experimental setting, LEAP2 (1–14) changed the FRET signal in a similar fashion [Proximity ratio change of GHSR and D2R = 11.86 ± 1.04 for LEAP2 (1–12) and 13.32 ± 1.13 for LEAP2 (1–14), Student’s *t* test, *p* = 0.3949, *n* = 3 each], indicating that both peptides have the same impact on GHSR conformation. Thus, the effect of N-terminal LEAP2 on the conformational features of GHSR is independent of GHSR-D2R heteromer presence.

We previously showed that GHSR is preassembled to Gα_q_ in the GHSR-D2R complex (Damian et al., 2018). To test if LEAP2 affects this preassembly, we monitored the FRET signal between Gα_i1_ and Gα_q_ in lipid nanodiscs containing GHSR and D2R. Dopamine triggered a significant FRET signal between Gi and Gq (Figure 4C), suggesting that dopamine recruits Gi to GHSR-D2R heteromers where Gq is preassembled, as previously reported (Damian et al., 2018). In contrast, dopamine failed to induce FRET signal in the presence of LEAP2 (1–12) or SPA, suggesting that both GHSR inverse agonists dissociate the GHSR-Gq preassembled complex, consistent with our previous observations with monomeric GHSR and SPA (Damian et al., 2018).

Finally, we tested whether LEAP2 (1–12) modifies the effect of GHSR on dopamine-evoked Gi protein activation using isolated GHSR-D2R heteromers in lipid nanodiscs. Specifically, we measured Gi activation by monitoring the rate of association of GTPγS to Gα_i1_βγ through the changes in Trp emission that accompanies GTPγS binding to Gα_i_. We incubated the lipid nanodiscs containing GHSR-D2R heteromers, Gα_i1_βγ and Gα_q_βγ with dopamine, in the absence or presence of LEAP2 (1–12), and measured the GTPγS association to Gα_i_ rate. We found that LEAP2 and SPA reverted the effect of GHSR on the kinetics of Gi activation, *i.e.,* the rate of GTPγS binding to Gα_i_ in the presence of dopamine and LEAP2 (1–12) was similar to that observed for dopamine to the D2R homomer (Figure 4D). These observations suggest that LEAP2 (1–12) abolishes the effect of GHSR-D2R association on dopamine-mediated Gi activation.

## Discussion

LEAP2 was recently recognized as an endogenous ligand of GHSR and shown to act as a receptor antagonist (Ge et al., 2018). Soon after, we and others showed that LEAP2 also acts as a GHSR inverse agonist (M'Kadmi et al., 2019; Wang et al., 2019). Here, we show that LEAP2 antagonizes the ghrelin-evoked inhibition of Ca_V_2.2, which involves Gq protein signaling (Lopez Soto et al., 2015). We also found that LEAP2 impairs the basal reduction of Ca_V_2.2 currents induced by constitutive GHSR activity, which involves Gi/o protein activation (Lopez Soto et al., 2015). These observations are in line with those where LEAP2 reduces not only ghrelin-evoked Gq protein signaling but also the ligand-independent Gq, Gi/o and G12/13 signaling recruited by GHSR (M'Kadmi et al., 2019). Thus, the binding of LEAP2 to GHSR displays a number of effects that result in a reduction of both ghrelin-dependent and ghrelin-independent modes of GHSR action.

The current finding showing that LEAP2 impairs the actions of GHSR on Ca_V_2.2 has important implications for the control of neuronal activity. We have shown that ghrelin-dependent and ghrelin-independent activities of GHSR impair native presynaptic Ca_V_2.2 currents and reduce GABA release from hypothalamic and hippocampal neurons (Cabral et al., 2014; Lopez Soto et al., 2015; Martinez Damonte et al., 2018). Such presynaptic effects of GHSR result in disinhibition of post-synaptic neurons and contribute to enhance GHSR mediated neuronal activation due to other molecular mechanisms, such as neuronal depolarization induced by ghrelin-mediated inhibition of voltage-gated potassium channels (Shi et al., 2013) and ghrelin-dependent and ghrelin-independent increase of AMPA receptor trafficking in hippocampal neurons (Ribeiro et al., 2014; Ribeiro et al., 2021). LEAP2 inhibition of GHSR activity converts this peptide into a putative important player in the control of neuronal plasticity and excitability. In this regard, LEAP2 prevents the ghrelin-induced depolarization of neuropeptide-Y-producing (NPY) neurons of the hypothalamic arcuate nucleus (Mani et al., 2019), a critical area for ghrelin-induced appetite (Luquet et al., 2005). Also, acute application of LEAP2 hyperpolarizes NPY neurons (Mani et al., 2019), suggesting that GHSR basally acts on these neurons and that LEAP2 impairs such activity. The precise molecular mechanisms engaged by LEAP2 to modulate the neuronal activity are unknown. Based on our data, we propose that regulation of Ca_V_2.2 currents contributes to this effects of LEAP2.

GHSR can interact with D2R, allowing not only a crosstalk between their signaling pathways but also a putative mutual allosteric regulation. In order to investigate whether LEAP2 affects GHSR modulation of D2R, we took advantage of the fact that GHSR dramatically impacts D2R inhibition of Ca_V_2.2 currents. The mechanisms by which D2R modulates Ca_V_2.2 channels are diverse. Dopamine-evoked D2R activation reduces: 1) Ca_V_2.2 currents through a membrane-delimited mechanism that depends on Gi/o protein in neostriatal cholinergic neurons (Yan et al., 1997) and 2) Ca_V_2.2 currents via voltage-dependent and voltage-independent mechanisms in a heterologous expression system (Kisilevsky and Zamponi, 2008). D2R was also shown to physically interact with Ca_V_2.2 and to control its traffic to the plasma membrane in a dopamine-independent manner (Kisilevsky and Zamponi, 2008). Recently, we showed that D2R reduces basal Ca_V_2.2 currents and that this reduction is prone to be removed by depolarization (Cordisco Gonzalez et al., 2020). Notably, we have shown that constitutive GHSR activity in GHSR-D2R heteromers alters basal and dopamine-evoked D2R inhibition of Ca_V_2.2 currents (Cordisco Gonzalez et al., 2020). In particular, we found a stronger reduction of basal Ca_V_2.2 current in presence of GHSR-D2R than in the presence of GHSR alone, and such effect requires Gq and Gβγ. On the other hand, dopamine has a smaller acute inhibitory effect on Ca_V_2.2 current in presence of GHSR-D2R than in presence of D2R alone, and the mechanism switches from partially Gβγ-dependent to an independent one. Based on these observations and considering that we have previously shown a close interaction between Gq and Gi/o coupled to GHSR and D2R respectively (Damian et al., 2018), we proposed a model in which GHSR sequestrates Gβγ dimers from Gi/o coupled to D2R (Cordisco Gonzalez et al., 2020). The downstream mechanism that reduces Ca_V_2.2 currents may implicate a membrane channel protein density reduction and/or a Gq-mediated basal inhibition of Ca_V_2.2 function*.* Here, we show that LEAP2 impaired the capability of GHSR to inhibit the basal and dopamine-evoked D2R-mediated reduction of Ca_V_2.2 currents, which also depends on ghrelin-independent Gq signaling coupled to GHSR. The actions of LEAP2 involve the N-terminal segment of the peptide (the region that binds to GHSR) and does not require its C-terminal portion, suggesting that LEAP2 does not physically disrupt GHSR-D2R interaction. In line with this possibility, we found here that LEAP2 does not dissociate the assembly of the GHSR-D2R heteromers, although it likely affects its arrangement and/or dynamics.

Early studies showed that GHSR shifts the dopamine-evoked and basal signaling of D2R to a non-canonical Gi/o protein signaling, independent of ghrelin-evoked and constitutive GHSR activity (Kern et al., 2012). We previously proposed that such GHSR-mediated shift in D2R signaling mechanism could be due to an allosteric effect of Gq on D2R-induced Gi activation when the former was preassembled to GHSR (Damian et al., 2018). Interestingly, the preassembly of GHSR to Gq does not occur when the receptor is stabilized in its inactive conformation upon binding of SPA (Damian et al., 2015). We show here that LEAP2 stabilizes the same inactive conformation of GHSR when this receptor is associated to D2R. Hence, a possible model would be that the N-terminal region of LEAP2 abolishes the preassembly of GHSR to Gq because it stabilizes an inactive state of the receptor within dimeric assembly. Alternatively, the effects of LEAP2 on GHSR-D2R-mediated regulation of Ca_V_2.2 currents could be related to changes in the heteromer’s organization or in the protomer exchange dynamics triggered by LEAP2, as such changes were also experimentally observed.

Interestingly, LEAP2 and ghrelin display similar binding affinities for GHSR (Ge et al., 2018; M'Kadmi et al., 2019; Wang et al., 2019), but plasma LEAP2 levels are ∼10-fold higher than plasma ghrelin levels in satiated rodents and humans (Mani et al., 2019; Fittipaldi et al., 2020). Thus, modulatory actions of LEAP2 on GHSR, such as those revealed here, may play a more dramatic role than ghrelin itself in some physiological GHSR functions (Cornejo et al., 2021). On the other hand, plasma ghrelin mainly acts on brain targets near the fenestrated capillaries, such the hypothalamic arcuate nucleus or the area postrema (Schaeffer et al., 2013; Cabral et al., 2014; Cabral et al., 2017). The observation that ghrelin displays a restricted accessibility to the brain has highlighted the notion that GHSR plays important ghrelin-independent actions in brain areas that are distantly located from fenestrated capillaries (Cabral et al., 2015; Perello et al., 2019). In this regard, abudant evidence shows that ghrelin-independent GHSR signaling in the mesolimbic pathway and hippocampus modulates different reward-related behaviors and learning/memory functions, respectively [as reviewed in Cornejo et al. (2020)]. Notably, we have found that the central administration of LEAP2 reduces binge-like intake of high-fat diet in mice (Cornejo et al., 2019). The molecular mechanisms by which LEAP2 affects the rewarding aspects of eating are uncertain. The fact that D2R plays a major role in brain regions involved in reward-related behaviors raises the possibility that LEAP2 regulation of GHSR-D2R heteromers impacts on high-fat intake.

## Conclusion

Our results provide detailed molecular insights that contribute to the ongoing efforts to clarify the mechanisms mediating LEAP2 actions. We show that LEAP2 not only has a dual action on GHSR, functioning as both an antagonist and as an inverse agonist, but also impairs GHSR regulation of D2R signaling. Importantly, GHSR forms heteromers with several other GPCRs, including the serotonin, oxytocin, orexin and non-D2R dopamine receptors. Thus, it is plausible to propose that the current observations represent a more general mechanism by which LEAP2 acts in the central nervous system.

## Data Availability

The original contributions presented in the study are included in the article/Supplementary Material, further inquiries can be directed to the corresponding authors.
